# Parabolic-Index Ring-Core Fiber Supporting High-Purity Orbital Angular Momentum Modes

**DOI:** 10.3390/s23073641

**Published:** 2023-03-31

**Authors:** Yuanpeng Liu, Yingning Wang, Wenpu Geng, Wenqian Zhao, Hao Zhang, Weigang Zhang, Zhongqi Pan, Yang Yue

**Affiliations:** 1 Institute of Modern Optics, Nankai University, Tianjin 300350, China; 2 Department of Electrical & Computer Engineering, University of Louisiana at Lafayette, Lafayette, LA 70504, USA; 3 School of Information and Communications Engineering, Xi’an Jiaotong University, Xi’an 710049, China

**Keywords:** orbital angular momentum, graded index, mode purity, ring fiber

## Abstract

We design a graded-index ring-core fiber with a GeO_2_-doped silica ring core and SiO_2_ cladding. This fiber structure can inhibit the effect of spin-orbit coupling to mitigate the power transfer among different modes and eventually enhance the orbital angular momentum (OAM) mode purity. By changing the high-index ring core from the step-index to parabolic graded-index profile, the purity of the OAM_1,1_ mode can be improved from 86.48% to 94.43%, up by 7.95%. The proposed fiber features a flexible structure, which can meet different requirements for mode order, effective mode area, etc. Simulation results illustrate that the parabolic-index ring-core fiber is promising in enhancing the OAM mode purity, which could potentially reduce the channel crosstalk in mode-division-multiplexed optical communication systems.

## 1. Introduction

A key issue in modern optical fiber communication systems is how to meet the rapidly growing traffic demand. To satisfy the explosive requirement for data bandwidth, extensive research has been conducted on multiplex optical signals in diverse photon dimensions, such as time, wavelength, amplitude, phase, polarization, and space [1,2,3,4,5]. According to Shannon’s theorem, there is an upper bound to the capacity in single-mode fiber transmission systems, which is a function of the available bandwidth and the signal-to-noise ratio of the link. With the continuous development of wavelength-division, polarization-division, and other multiplexing technologies, the capacity of communication systems has gradually approached the Shannon limit, and researchers have tried to implement other physical dimensions of the data signal to increase the transmission capacity and improve the spectral efficiency. Spatial division multiplexing (SDM) utilizes the degree of freedom of light waves in the physical dimension of space, which can be applied in multi-core fibers, multi-mode fibers, or multi-core multi-mode fibers to further improve the transmission capacity of optical fiber communication systems [6,7,8,9,10].

The SDM technology can be achieved in a multi-core fiber where each core works as a spatially independent channel for transmission, or in a multi-mode fiber where mutually orthogonal modes transmit different data, or in a multi-core multiple-mode fiber where greater transmission capacity in a single fiber is achieved by combining spatial multiplexing with mode multiplexing. Through the multiplexing over space and mode, the SDM technology can increase the communication capacity by one to two orders of magnitude, which has been one of the mainstream research and development directions of ultra-large-capacity optical fiber communication technology [11,12]. Currently, a scalar modal set composed of several eigenmodes, the linearly polarized (LP) modes, are widely used in SDM systems because of the simple excitation condition. However, a random mode coupling among the channels will arise during propagation because of fabrication imperfections or bending, which can introduce unwanted crosstalk between the optical modes. Nevertheless, the detrimental effect of mode coupling can be fully compensated for using a multiple-input multiple-output (MIMO) digital signal processing (DSP) algorithm, which could extremely increase the complexity and cost of the communication system. Different methods to reduce the use of MIMO processing have been investigated. One feasible scheme is mode-group multiplexing, which employs low differential group delays (DGD) and/or DGD management to lower the complexity of MIMO DSP [13,14,15,16]. Moreover, polarization-maintaining optical fibers, for example, the elliptical-core fibers, are also adopted for MIMO-less SDM, in which all the degeneracies among the vectorial modes are broken [17,18].

In addition to the above schemes, the multiplexing technique of beams carrying orbital angular momentum (OAM) offers a new direction for simplifying communication systems [19,20]. OAM is a natural property of a beam with a helical phase and, theoretically, has infinite topological charges. The helical phase front of a beam carrying OAM can be described as e*^ilφ^*, where *φ* is the azimuth angle and *l* is the topological charge [21]. OAM modes with different topological charges are mutually orthogonal, so different OAM modes can provide a new degree of freedom for multiplexing technology. Furthermore, the multiplexing of OAM is independent of polarization and wavelength, which makes it have significant potential in improving the capacity of optical fiber communication systems [22,23,24,25,26]. With proper design, different OAM modes transmitted in the fiber can have a large index separation to mitigate the modal crosstalk. Compared with the traditional LP modes, it will have a smaller inter-mode crosstalk, which means it can be used to lessen the demand on the MIMO system [16,20,27].

To achieve stable transmission of OAM beams, limit the generation of unwanted radially high-order modes, and match their doughnut-shaped optical field distributions, various ring-core fibers (RCFs) have been proposed [28]. In 2009, Ramachandran et al. found that the annular high-refractive-index region in the fiber was adapted to the intensity profile of the vector beam, allowing the vector beam to maintain better stability and purity [29]. In 2013, the same kind of ring-core fiber was used to transmit OAM modes. This kind of vortex fiber was used to simultaneously couple two OAM modes with *l* = ±1 and two polarization-multiplexed fundamental modes [20]. In 2014, C. Brunet et al. designed and fabricated a ring fiber that is capable of transmitting 36 OAM modes [30]. In 2015, an air-core fiber was reported to further increase the refractive index difference between eigenmodes, which could increase the number of OAM modes supported in the fiber [31]. However, the large refractive index contrast between the air core and the high-index ring will greatly enhance the spin-orbit coupling (SOC) effect in the fiber, so that the OAM carried by a portion of the transmitted beam will be converted to spin angular momentum (SAM), which means the transmitted vortex light is no longer a pure circularly polarized OAM mode. Thus, the purity of the mode will be reduced, leading to the reduction in the demultiplexing efficiency of the orthogonal OAM modes at the receiving end and the increase in the crosstalk between modes [32,33,34,35]. Therefore, finding a proper solution for the problem of the reduced purity of the OAM modes can provide a certain reference for the design and manufacturing of OAM fibers in the future. Ref. [36] proposed a dual-step-index RCF structure to suppress the spin-orbit effect and, thus, improve the OAM mode purity. Similar to Ref. [36], the graded-index profile is also a promising solution, which can suppress the SOC by reducing the abrupt refractive index change. In recent years, some researchers have used the graded-index profile (GIP) in their proposals. Nevertheless, the related fibers in these works either are not the ring-core fiber [37], which cannot match the mode field distribution of the OAM modes well, or have complicated structures that may be challenging for the current fabrication technology [38]. Therefore, it is necessary to systematically analyze the characteristics of the OAM modes in the graded-index ring-core fiber (GIRCF). Conducting this kind of research would, thus, be beneficial to further understand the effect of the refractive index profile on the OAM mode properties.

In this paper, we design a graded-index ring-core fiber and investigate the properties of its supported OAM mode. By adopting a parabolic-index profile to reduce the abrupt index change between the ring core and the cladding, the SOC effect can be restrained at the boundary between the ring core and cladding. The power of the transmitted OAM mode can be well-maintained in its initial mode order, and the corresponding mode purity can be thus improved. At 1550 nm, the GIRCF can support up to a total of 322 OAM modes. Additionally, the purity of the first-order OAM mode in GIRCF is notably higher than that of the step-index ring core fiber, which can be increased from 86.48% to 94.43%.

## 2. Concept and Fiber Structure

### 2.1. Fiber Structure

**Figure 1:**
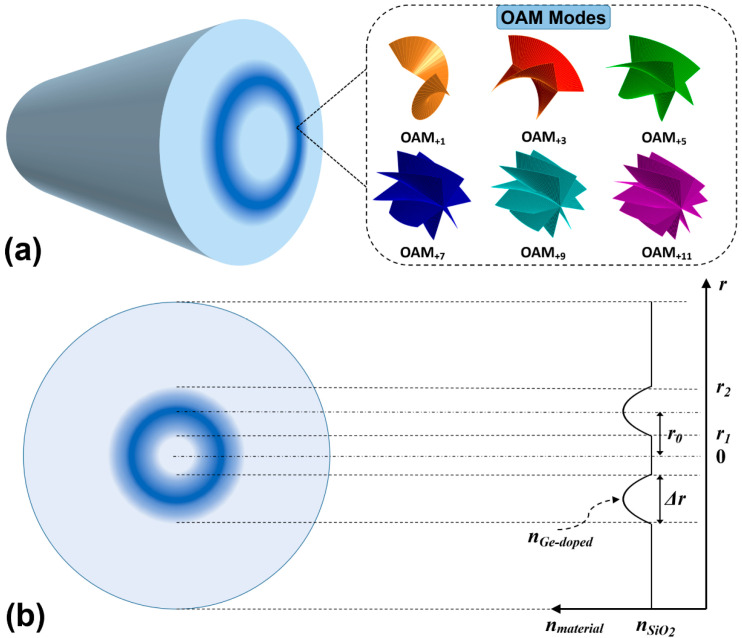
(**a**) Concept of OAM mode-division multiplexing in the proposed GIRCF; (**b**) cross-section view and refractive index profile of the proposed GIRCF.

Figure 1a illustrates the concept of multiplexing OAM modes with different orders in a GIRCF, and Figure 1b depicts the cross-section and index profile of the designed GIRCF. As shown in Figure 1b, the GIRCF is composed of a high-index GeO_2_-doped silica ring core and SiO_2_ background. The refractive index profile in the high-index ring-core region can be expressed as:

(1) $$n(r)=\left\{ \begin{array}{ll} n_{1}\times \sqrt{1-\frac{n_{1}^{2}-n_{2}^{2}}{n_{1}^{2}}\times {\left(\frac{2\left|r-r_{0}\right|}{\Delta r}\right)}^{\alpha }} & ;r_{1}<r_{2}\\ n_{2} & ;0\leq r\leq r_{1}\;or\;r\geq r_{2} \end{array}\right.$$

where *n*_1_, *n*_2_, *r*_0_, Δ*r*, and *α* are the material index of the GeO_2_-doped ring core, the material index of the background silica, the radius from the fiber center to the ring center, the thickness of the ring-core region, and the shape factor, respectively. The refractive indices of the 5% GeO_2_-doped silica and silica (SiO_2_) at 1550 nm are 1.4515 and 1.444, respectively, and the corresponding relative refractive index difference (Δ) is 0.52% [39]. Their wavelength dependence is considered by using the Sellmeier equations [40]. In GIRCF, *r*_0_ represents the position of the ring core, which can be adjusted from 7 μm to 47 μm to afford more eigenmodes, and the shape factor *α* controls the gradient of the refractive index between the high-index ring core and cladding. When *α* is equal to 2, the refractive index profile of the ring-core region is parabolic. As *α* increases, the upper and lower edges of the parabola will become steeper. When *α* approaches infinity, the refractive index profile will become a step-index one. The smaller the *α* is, the slower the refractive index transition between the ring core and cladding is. It can inhibit the coupling effect of SAM and OAM, which is a form of spin-orbit interaction at the ring-core–cladding boundary. Therefore, the power of the OAM mode transmitted in the designed fiber will not couple to the other-order OAM modes, which is beneficial to enhance the OAM mode purity. The thickness of the ring (Δ*r*) also needs proper design to avoid radially high-order modes [41]. All numerical analyses and simulated calculations of the designed fiber are performed using the finite element method (FEM) in COMSOL Multiphysics 5.4 software.

### 2.2. Fabrication Feasibility of the GIRCF

During the past decade, researchers have fabricated a variety of ring-core fibers. The mole fraction of the GeO_2_ in the fiber changes from 5% to almost 30% [30,42,43,44]. Highly Ge-doped fiber core with GeO_2_ content up to 98 mol% has also been manufactured in practice [45]. Moreover, due to the limitations of the current manufacturing technology, the fabricated step-index ring-core fiber (SIRCF) often presents a GIP [28]; thus, it is feasible to fabricate the investigated GIRCF using modified chemical vapor deposition (MCVD) or plasma chemical vapor deposition (PCVD) [46,47]. The following study comprehensively considers the fabrication possibilities and analyzes the OAM mode characteristics in the GIRCF under different GeO_2_ mole fractions.

### 2.3. OAM Modes Supported in the GIRCF

OAM modes in the GIRCF can be expressed as a superposition of two same-order eigenstates with the same propagation constants:

(2) $$OAM_{\pm l,m}^{\pm }=HE_{l+1,m}^{even}\pm jHE_{l+1,m}^{odd}$$

(3) $$OAM_{\pm l,m}^{\mp }=EH_{l-1,m}^{even}\pm jEH_{l-1,m}^{odd}$$

where *l* is the topological charge, |*l*| is the OAM mode order, *m* is the radial index, and j represents the phase shift of π/2 between the even and odd modes. The HE and EH in Equations (2) and (3) represent two fundamental eigenstates of the OAM modes. As the radially high-order modes are undesirable, we take *m* = 1 in our designed fiber. The superscripts “±” represent the right-hand or left-hand circular polarization direction, and the subscripts “±” represent the right or left rotation direction of the OAM mode phase front, respectively. One of the OAM mode properties is the unlimited topological charge number, so the value of *l* can theoretically be any integer. In general, the OAM_0,1_ mode composed by HE_1,1_ is not considered as standard OAM because its phase front is not helical.

## 3. OAM Mode Properties

### 3.1. Intensity and Phase Distributions

**Figure 2:**
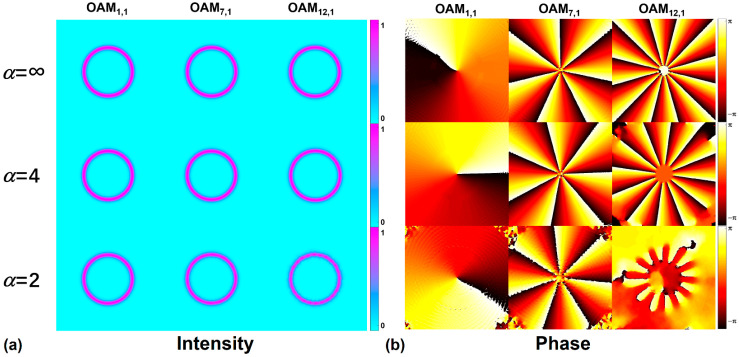
(**a**) Intensity and (**b**) phase distributions of the OAM_1,1_, OAM_7,1_, and OAM_12,1_ modes in the RCF with *α* = 2, 4, ∞, respectively.

As shown in Figure 2, we compare the normalized intensity and phase distributions of OAM modes (*l* = 1, 7, 12) under different shape factors (*α*) in RCF. Different OAM modes in the figure are composed of the coherent superposition of the even and odd fiber eigenmodes of HE_2,1_, HE_8,1_, and HE_13,1_. As one can see from the figure, when the refractive index profile evolves from a step to a parabolic shape, the intensity distributions of the corresponding OAM modes are still well-confined in the ring region, and the phase distribution of the OAM*_l_*_,1_ mode has a 2*l*π change azimuthally.

### 3.2. Supported OAM Mode Number and the Effective Refractive Indices

**Figure 3:**
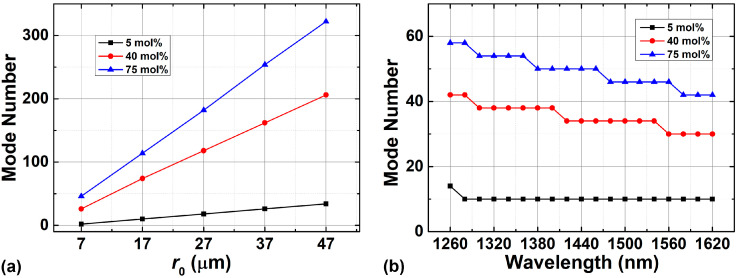
OAM mode number supported in the fiber as a function of (**a**) the position of the ring-core (*r*_0_) and (**b**) wavelength with different mole fractions of GeO_2_.

The supported OAM mode number in RCFs can be calculated as [48]:

(4) $$n_{\mathrm{OAM}}=(n_{\mathrm{HE}}-1+n_{\mathrm{EH}})\times 2$$

where *n*_OAM_, *n*_HE_, and *n*_EH_ stand for the OAM mode number, the HE eigenmode number, and the EH eigenmode number supported in RCFs, respectively. Figure 3 displays the maximum number of OAM modes supported in the fiber as a function of the ring-core position and wavelength under different GeO_2_ mole fractions when the thickness of the ring-core region is 1.93 μm and the shape factor equals 2. The high refractive index area expands with the increasing radius of the ring-core region (*r*_0_), when the ring-core thickness (Δ*r*) is fixed. As a result, the number of modes supported in GIRCF monotonically increases as the ring-core position moves out, as shown in Figure 3a. When *r*_0_ equals 47 μm and the GeO_2_ mole fraction is 5 mol%, the designed GIRCF is capable of supporting up to 34 modes. In addition, Figure 3b indicates that the maximum number of OAM modes supported by GIRCF decreases as the wavelength increases because the higher-order modes will gradually leak out because of the shorter cut-off wavelength.

**Figure 4:**
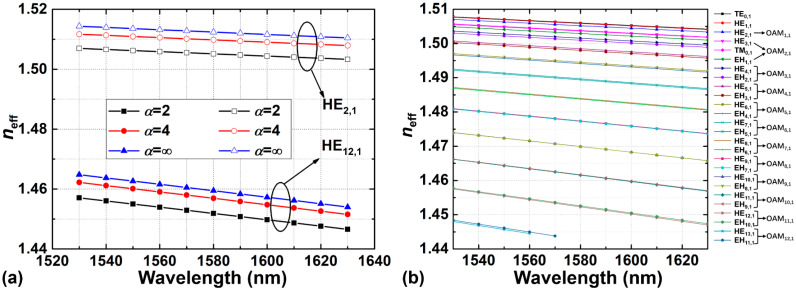
(**a**) Effective refractive indices of the HE_2,1_ and HE_12,1_ modes with *α* = 2, 4, ∞; (**b**) effective refractive indices of the eigenmodes when *α* = 2.

Figure 4a shows the effective refractive indices (*n*_eff_) as a function of wavelength for the highest-order HE_12,1_ and lowest-order HE_2,1_ mode supported in the C + L band with 75 mol% GeO_2_ in the corresponding ring-core fibers under different shape factors (*α* = 2, 4, ∞) when *r*_0_ = 7 μm. The designed GIRCF can support the highest-order HE_12,1_ mode across the C + L band. Compared with the SIRCF, the effective refractive indices of OAM modes in the GIRCF will be lower, which means that some high-order modes (such as HE_13,1_) in the GIRCF will be cut off earlier with increasing wavelength. Under the same condition, Figure 4b illustrates the effective refractive indices of all vector eigenmodes supported in the GIRCF as a function of wavelength, in which the *n*_eff_ of the eigenmodes decreases with the increasing wavelength. We also calculate the effective index difference Δ*n*_eff_ between the HE eigenmodes and EH eigenmodes, which synthesize the same-order OAM modes. The calculated results indicate that Δ*n*_eff_ is larger than 1 × 10^−4^ in most cases, except for the ones of OAM_8,1_ and OAM_9,1_. The maximum and minimum Δ*n*_eff_ of the eigenmodes in the designed fiber is 7.78 × 10^−4^ for OAM_2,1_ and 4.82 × 10^−5^ for OAM_8,1_, respectively, which suggests a good performance in the modal separation.

### 3.3. Effective Mode Field Area (A_eff_) of the OAM Modes

**Figure 5:**
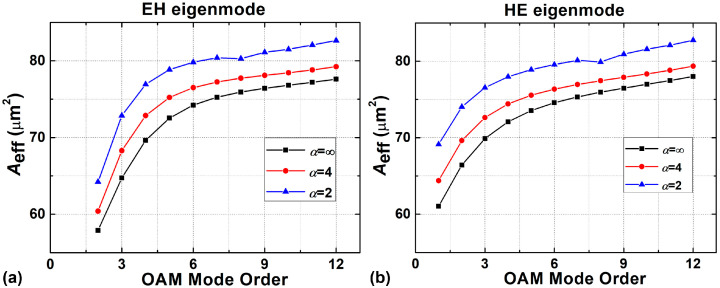
Effective areas of (**a**) the EH-composed OAM modes and (**b**) the HE-composed OAM modes in the ring-core fiber with *α* = 2, 4, ∞.

The effective mode area (*A*_eff_) as a function of the mode order is shown in Figure 5 with different shape factors (*α* = 2, 4, ∞) under the radially single-mode condition. *A*_eff_ is defined as [49]:

(5) $$A_{\mathrm{eff}}=\frac{{\left(\iint {\left|E(x,y)\right|}^{2}dxdy\right)}^{2}}{\iint {\left|E(x,y)\right|}^{4}dxdy}$$

where *E*(*x*, *y*) is the electrical field distribution of the transverse mode field.

**Figure 6:**
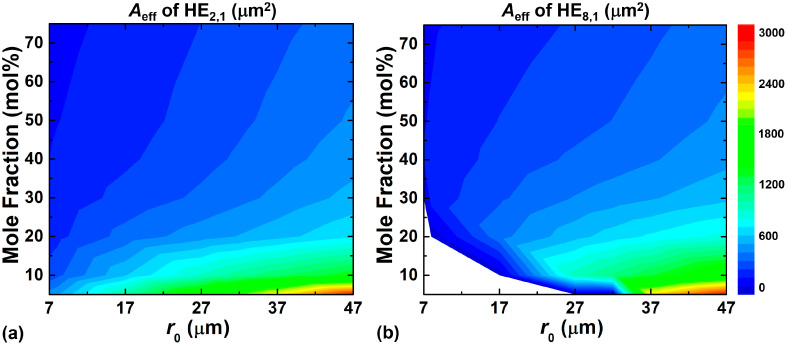
Effective areas of the (**a**) HE_2,1_ and (**b**) HE_8,1_ modes with *α* = 2 under different mole fractions of GeO_2_ and the position of the ring-core.

As can be seen from Figure 5, the GIRCF has a larger mode effective area than the SIRCF because of the weaker confinement of the mode field in GIRCF, and the effective mode area generally increases with the OAM mode order. Figure 6 depicts the effective mode area of HE_2,1_ and HE_8,1_ in GIRCF with a parabolic index profile and a 1.93 μm ring width as a function of the ring-core position under different mole fractions of GeO_2_, respectively. It is clear that the effective mode area increases as the ring-core position moves out and the mole fraction of GeO_2_ decreases. The blank area in Figure 6b means that the HE_8,1_ mode has been cut off. To evaluate the ability of the GIRCF to suppress the nonlinear effects, we analyze the nonlinear coefficient *γ* of the fiber under different GeO_2_ mole fractions. The calculation results show that the values of *γ* are 2.1 × 10^−2^/W/km, 1.4/W/km, and 2.8/W/km when the GeO_2_ mole fractions are 5 mol%, 40 mol%, and 75 mol%, respectively [50]. According to Ref. [51], the value of *γ* in traditional single mode fiber is 1.267/W/km. When the mole fraction is up to 40 mol%, the value of *γ* in GIRCF is still close to the one in SMF, which means they have similar nonlinearity-limited transmission characteristics. Consequently, with a lower GeO_2_ mole fraction, the GIRCF could potentially support a longer transmission distance.

### 3.4. Mode Purity

In standard optical fibers, the degeneracy of OAM eigenstates is lifted because of the spin-orbit-interaction-induced effective index splitting, which causes the instability of the OAM eigenstates in practice. The ring-core fiber designs can support OAM modes with an order-of-magnitude larger effective refractive index difference, which facilitates stable transmission of a large number of OAM modes. However, the larger refractive index difference also introduces strong SOC. Therefore, the weakly guiding approximation is not considered in the formula of OAM synthesis. In the strong SOC case, the beams propagating in the fiber are the superpositions of the SAM and OAM states [52]. Consequently, the exact solution of the waveguide characteristic equation will not be a single OAM mode, i.e., the synthesized OAM mode is not pure when a large refractive index discontinuity exists at the interface between the ring core and cladding. Part of the power of the *l*^th^-order OAM mode transmitted in fiber could be coupled into the other-order OAM modes. Nevertheless, the GIP can greatly alleviate the discontinuous area, thus effectively inhibiting the SOC. Consequently, the GIRCF can enhance the purity of the OAM modes and diminish the intrinsic crosstalk by suppressing the spin-orbit effect at the high-contrast interface. We define the ratio of the dominant component power to the whole power transmitted in the fiber as OAM mode purity, which can be written as [33]:

(6) $$\mathrm{Purity}=\frac{power_{OAM_{major}}}{power_{OAM_{major}}+power_{OAM_{minor}}}$$

**Figure 7:**
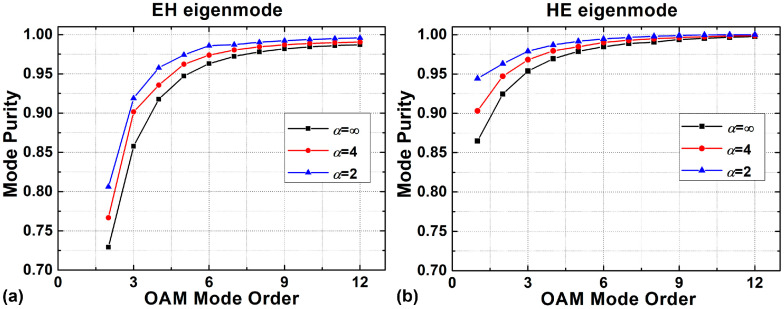
Purity of the (**a**) EH-composed and (**b**) HE-composed OAM modes with different shape factors.

Figure 7 illustrates the relationship between the purity of the EH-based and HE-based OAM modes with different shape factors under a radially single-mode condition. The purity of the OAM mode coherently synthesized by the HE eigenmodes is generally higher than that synthesized by the EH eigenmodes with the same order, and higher-order OAM modes have better performance in purity. It should be noted that the GIRCF has higher mode purity than the SIRCF under the same radially single-mode condition. Furthermore, the GIRCF with a smoother refractive index profile has a higher mode purity, which can be attributed to the inhibition of GIP on SOC. In addition, the mode purity of HE-composed OAM_1,1_ in GIRCF reaches up to 94.43%, increasing by 7.95% over the one in the SIRCF, and the purity of EH-composed OAM_2,1_ mode increases by 7.70%. This means that the purity of low-order OAM modes has a significant improvement in the GIRCF. According to Ref. [33], the intrinsic crosstalk can be expressed as:

(7) $$Crosstalk=10\times \log_{10}\left(1-purity\right)$$

when the purity is improved from 86.48% to 94.43%, the crosstalk can be reduced from −8.69 dB to −12.54 dB. Moreover, under ideal circumstances, when the OAM mode purity is larger than 96.84%, the system crosstalk can be smaller than −15 dB, which means the transmission system can be a MIMO-free system [20].

**Figure 8:**
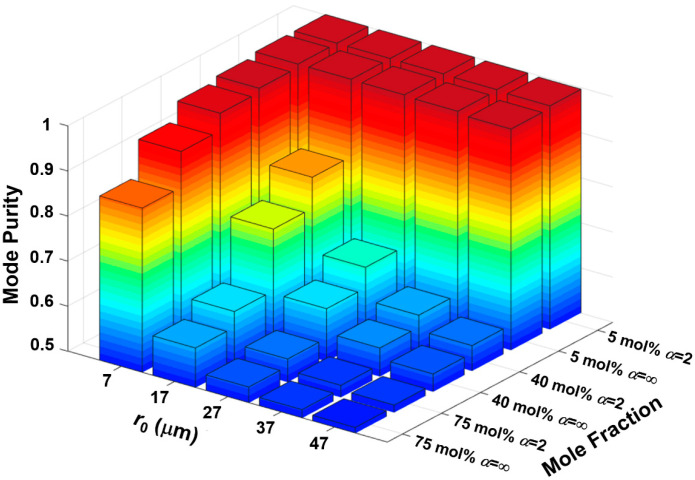
OAM mode purity based on HE_2,1_ eigenmodes as a function of the position of ring-core for different GeO_2_ mole fractions.

To further observe the effect of spin-orbit interaction and verify the purity enhancement by alleviating the index discontinuity, we calculate and compare the mode purity of HE-composed OAM_1,1_ with different GeO_2_ mole fractions and ring-core positions under different shape factors, as shown in Figure 8. As mentioned above, the high GeO_2_ mole fraction will introduce strong SOC, which means that the effect of GIP on SOC will be particularly obvious. Therefore, we can see that the purity of the OAM_1,1_ can be promoted significantly under a higher GeO_2_ mole fraction.

**Figure 9:**
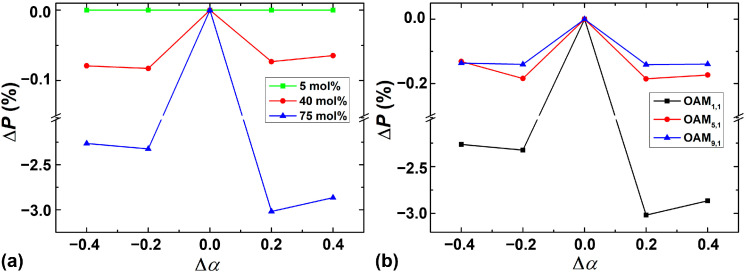
Purity deviation Δ*P* as a function of Δ*α* with (**a**) different mole fractions of GeO_2_ for OAM_1,1_ mode, and (**b**) different OAM modes from OAM_1,1_ to OAM_9,1_ for 75 mol% GeO_2_ mole fraction.

Considering the defects introduced into the index profile during the fabrication process, a perturbation type of calculation for shape factor *α* is analyzed when *r*_0_ = 7 μm. The purity deviation of the OAM mode (Δ*P*), as a function of shape factor error (Δ*α*) from *α* = 2, is displayed in Figure 9. The mode purity for *α* = 2 is used as the reference, and Δ*P* represents the deviation between the OAM mode purity under *α* + Δ*α* and the reference. Figure 9a shows that Δ*P* of OAM_1,1_ varies with Δ*α* under three GeO_2_ mole fraction cases, and Figure 9b depicts that Δ*P* of different OAM modes varies with Δ*α*. As shown in Figure 9a, under the 5 mol% and 40 mol% GeO_2_ mole fractions, the deviation of the OAM mode purity could be <0.1% when the shape factor error is within ±0.4, respectively. In addition, the deviation of the OAM mode purity could be <0.2% in high-order OAM modes when the GeO_2_ mole fraction is 75 mol%. Therefore, by selecting proper GeO_2_ concentration and OAM modes with higher order, the fabrication tolerance could be further increased.

## 4. Conclusions and Perspective

In this paper, we design a GIRCF having a GeO_2_-doped silica ring core and SiO_2_ background. This fiber can restrain the effect of spin-orbit interaction, thus improving the mode purity of OAM modes transmitted in fiber. The GIRCF can support up to a total of 322 OAM modes at 1550 nm. Compared with SIRCF, the modes supported in the GIRCF have different degrees of enhancement in the mode purity, in which the maximum purity improvement of the HE-composed OAM_1,1_ reaches up to 7.95% from 86.48% to 94.43%. Moreover, by changing the position of the ring core and the mole fraction of the GeO_2_, the designed GIRCF can satisfy different requirements for SDM. This kind of fiber is promising in the enhancement of OAM mode purity, which can decrease the intrinsic channel crosstalk in optical fiber communication.

## Data Availability

Data underlying the results presented in this paper are not publicly available at this time but may be obtained from the authors upon reasonable request.
